# Factors contributing to delay in parasite clearance in uncomplicated falciparum malaria in children

**DOI:** 10.1186/1475-2875-9-53

**Published:** 2010-02-15

**Authors:** Akintunde Sowunmi, Elsie O Adewoye, Grace O Gbotsho, Christian T Happi, Abayomi Sijuade, Onikepe A Folarin, Titilope M Okuboyejo, Obaro S Michael

**Affiliations:** 1 Department of Pharmacology & Therapeutics, University of Ibadan, Ibadan, Nigeria; 2 Malaria Research Laboratories, Institute for Medical Research and Training, University of Ibadan, Ibadan, Nigeria; 3 Department of Physiology, University of Ibadan, Ibadan, Nigeria

## Abstract

**Background:**

Drug resistance in *Plasmodium falciparum *is common in many endemic and other settings but there is no clear recommendation on when to change therapy when there is delay in parasite clearance after initiation of therapy in African children.

**Methods:**

The factors contributing to delay in parasite clearance, defined as a clearance time > 2 d, in falciparum malaria were characterized in 2,752 prospectively studied children treated with anti-malarial drugs between 1996 and 2008.

**Results:**

1,237 of 2,752 children (45%) had delay in parasite clearance. Overall 211 children (17%) with delay in clearance subsequently failed therapy and they constituted 72% of those who had drug failure, i.e., 211 of 291 children. The following were independent risk factors for delay in parasite clearance at enrolment: age less than or equal to 2 years (Adjusted odds ratio [AOR] = 2.13, 95% confidence interval [CI]1.44-3.15, P < 0.0001), presence of fever (AOR = 1.33, 95% CI = 1.04-1.69, P = 0.019), parasitaemia >50,000/ul (AOR = 2.21, 95% CI = 1.77-2.75, P < 0.0001), and enrolment before year 2000 (AOR= 1.55, 95% CI = 1.22-1.96, P < 0.0001). Following treatment, a body temperature ≥ 38°C and parasitaemia > 20000/μl a day after treatment began, were independent risk factors for delay in clearance. Non-artemisinin monotherapies were associated with delay in clearance and treatment failures, and in those treated with chloroquine or amodiaquine, with *pfmdr 1/pfcrt *mutants. Delay in clearance significantly increased gametocyte carriage (P < 0.0001).

**Conclusion:**

Delay in parasite clearance is multifactorial, is related to drug resistance and treatment failure in uncomplicated malaria and has implications for malaria control efforts in sub-Saharan Africa.

## Background

The emergence and spread of multidrug resistance in *Plasmodium falciparum *is a major obstacle to successful chemotherapeutic control of the disease. Resistance to chloroquine (CQ) and sulphadoxine-pyrimethamine (SP) is now widespread in sub-Saharan Africa, South Asia and South America [1,2] and there is an increased chance that resistance to mefloquine (MQ), already widespread in Southeast Asia [3-6], may spread to Africa. With increasing failure of amodiaquine (AQ) in areas of intense transmission [7-9] and increasing selection of *P. falciparum *multidrug resistance gene 1 (*Pfmdr 1*) in asexual and sexual parasites following treatment of infections with artemether-lumefantrine (AL) [10], there is a rising spectre of reduced responses to artemisinin-based combination therapy (ACT) in Africa.

Despite increasing drug treatment failure, there is no clear guidelines, at least in Nigeria, about the time to change anti-malarial drug treatment if parasites do not clear quickly from peripheral blood following treatment of uncomplicated acute infections in African children. It is postulated in the present study that, parasite clearance exceeding two days is associated with risk of treatment failure and resistance and could be used as a criterion to change therapy in very young children. The present study reports the relationship between delay in parasite clearance and anti-malarial treatment failure in children with falciparum malaria in an area of intense transmission in south-western Nigeria, where resistance in *P. falciparum *to CQ and SP deteriorated steadily over the past ten years.

## Methods

### Patients

The studies took place between July 1996 and July 2008 in patients presenting at the University College Hospital in Ibadan, a hyperendemic area for malaria in south-western Nigeria [11]. Ethical clearance was provided by the local ethics committee. During the period, a series of anti-malarial drug studies were conducted to evaluate the efficacy and safety of different treatment regimens (Table 1). Details of the drug efficacy studies have been described before [12-16].

**Table 1:** Treatment regimens and time of study in the children enrolled

Drugs*	Regimens†	No of patients	Year
AQ	30 mg/kg of amodiaquine base over 3 days, that is, 10 mg/kg daily	573	2000-2006
AQAS	Artesunate given as 4 mg/kg dail for 3 d plus amodiaquine given as in AQ above	142	2004-2005
AQPS	Amodiaquine given as in AQ above plus sulphadoxine-pyrimethamine given as 25 mg/kg of the sulphadoxine component at presentation	69	2000
AQSP	Amodiaquine given as in AQ above plus sulphalene-pyrimethamine given as 25 mg/kg of the sulphalene component at presentation	91	2006
AMQ	Mefloquine given as 25 mg/kg at presentation plus artesunate as given in AQAS above	174	2007-2008
AL	Artemether (20 mg) plus lumefantrine (120 mg) given thus: 5-14 kg received 1 tab., 15-24 kg received 2 tab., 25-34 kg received 3 tab., > 34 kg received4 tab. at presentation, 8 h later and at 24, 36, 48 and 60 h after first dose	90	2006
AS	Artesunate given as 28 mg/kg over 7 days, that is, 4 mg/kg daily	120	2006
COT	Co-trimoxazole given as 20 mg of the sulphamethoxazole component twice daily	104	1998-1999
CQ	30 mg/kg of chloroquine base over 3 days, that is, 10 mg/kg daily	388	1996-2004
CQCP	30 mg/kg of chloroquine base over 3 days, that is, 10 mg/kg daily plus chlorpheniramine 8 mg start and 4 mg 8 hourly for 5 d.	315	1996-1999
CQKET	30 mg/kg of CQ base over 3 days, i.e., 10 mg/kg daily plus ketotifen 25 mg/kg statim, followed by 0.125 mg/kg 8 hourly for 4 d.	70	2001
CQPS	30 mg/kg of amodiaquine base over 3 days, that is, 10 mg/kg daily plus sulphadoxine-pyrimethamine given as 25 mg/kg of the sulphadoxine component at presentation	107	2000/2004
MQ	Mefloquine given as 25 mg/kg at presentation	176	2007-2008
SP	Pyrimethamine-sulphadoxine given as 25 mg/kg of the sulphadoxine component at presentation	291	1996-2004
SPP	Sulphadoxine-pyrimethamine given as in PS above plus probenecid at 20-25 mg/kg in two divided doses daily for 3 day	42	2004

† All drugs were administered orally. AQ, amodiaquine; AQART, amodiaquine plus artesunate; AQPS, amodiaquine plus pyrimethamine-sulphadoxine; AQSP, amodiaquine-sulphalene-pyrimethamine; ARTMF, mefloquine plus artesunate; ARTMLUM, artemether plus lumefantrine; AS, artesunate; COT, cotrimoxazole; CQ, chloroquine; CQCP, chloroquine plus chlorpheniramine; CQKET, chloroquine plus ketotifine; CQPS, chloroquine plus pyrimethamine-sulphadoxine; MQ, mefloquine; PS, pyrimethamine-sulphadoxine; PSP, pyrimethamine-sulphadoxine plus probenecid;

Briefly, children with symptoms compatible with acute falciparum malaria who fulfilled the following criteria were enlisted in the study: age 144 months or below, mono-infection with *P. falciparum*, parasitaemia ≥ 2,000 asexual forms/μl blood, negative urine tests for anti-malarial drugs 4-aminoquinolines and sulphonamides (Dill-Glazko and lignin tests, respectively), absence of concomitant illness, no evidence of severe malaria [17], and written informed consent given by parents or guardians. After enrolment and start of treatment (day 0), follow-up with clinical and parasitological evaluation was at days 1-7, and then on days 14 - 28 up to 2004. After 2004, follow-up was for 42 d. Clinical evaluation consisted of a general clinical examination including measurement of weight, core temperature and physical examination.

### Assessment of parasitaemia

Thick and thin blood films prepared from a finger prick were Giemsa-stained and were examined by light microscopy under an oil-immersion objective, at × 1,000 magnification, by two independent assessors. Parasitaemia in thick films was estimated by counting asexual parasites relative to 1,000 leukocytes, or 500 asexual forms, whichever occurred first. From this figure, the parasite density was calculated assuming a leukocyte count of 6,000/μl of blood. Gametocytes were also counted in thick blood films against 1,000 leukocytes assuming an average leukocyte count of 6,000/μl of blood [18-20]. Haematocrit was done at enrolment in 994 of the patients treated with non-artemisinin combination therapy (NACT) or ACT in order to evaluate the safety of combination anti-malarial therapies.

### Evaluation of response to drug treatment

Response to drug treatment was assessed using World Health Organization (WHO) criteria [21] as follows: S = sensitive, clearance of parasitaemia without recurrence; RI (mild resistance) = parasitaemia disappears but reappears within 7 to 14 days; RII (moderate resistance) = decrease of parasitaemia but no complete clearance from peripheral blood; RIII (severe resistance) = no pronounced decrease or increase in parasitaemia at 48 hours after treatment. In those with sensitive or RI response, parasite clearance time (PCT) was defined as the time elapsing from drug administration until there was no patent parasitaemia for at least 72 h. Delay in parasite clearance was defined as a parasite clearance time > 2 d, and was based on the asexual life cycle of 48 h in the infected erythrocyte [22]. The above criteria used for assessing responses to anti-malarials were used in order to maintain uniformity and because they were the recommended criteria when studies started.

### Molecular analysis of *pfmdr 1 *and *pfcrt*

At enrolment, 100 μl of capillary blood was collected on to 3 MM Whatman™ filter paper for resistance markers determination in 2000 and 2006 in those treated with CQ or AQ. *Pfmdr 1 *and *pfcrt *genotypes were assessed by PCR using methods described previously [7,10]. Single nucleotide polymorphisms in *pfmdr 1 *and *pfcrt *were detected by nested PCR-RFLP methods.

### Statistical analysis

Data were analysed using version 6 of the Epi-Info software [23], and the statistical program SPSS for Windows version 10.01 [24]. Proportions were compared by calculating χ^2 ^with Yates' correction. Normally distributed, continuous data were compared by Student's t-tests and analysis of variance (ANOVA). Data not conforming to a normal distribution were compared by the Mann-Whitney U-test and the Kruskal-Wallis test (or by Wilcoxon rank sum test). A multiple logistic regression model was used to test the association between parasite clearance > 2 d (yes or no at presentation or during follow up) and factors that were significant at univariate analysis: age, presence of fever, asexual parasitaemia at presentation or during follow-up, a history of vomiting, and drug treatment. Because the study was conducted over a period of 12 years, time in years since the commencement of trials was included as a covariate in the model for pretreatment delay in parasite clearance. P-values of < 0.05 were taken to indicate significant differences.

## Results

Between July 1996 and July 2008, 2,752 children (1342 females) were enrolled into the drug studies. All were recruited into prospective randomized studies and all had primary infections with *P. falciparum*. There were 1,716 under five-year olds. The geometric mean parasitaemia at enrolment was 34,044/μl (95% CI 30,400 - 33,664) (Table 2). Follow up was achieved in 2,562 children for up to 21 days and in 2,122 children for 28 or 42 days.

**Table 2:** Baseline clinical and parasitological parameters of the 2752 children enrolled in the study

Variables	Mean ± SD (range)	95% CI
Age (year)	6.1 ± 3.0 (0.5-12)	6.0-6.2
No. < 5 years	1716 (63%)	
Weight (kg)	17.3 ± 6.4 (5-47)	17.0-17.6
Axillary temperature (°C) (n = 2428)	38.3 ± 1.2 (34-42)	38.2-38.3
No. with > 40°C	210	
Haematocrit (%) (n = 994)	30.5 ± 4.8 (10-51)	30.1-30.7
No. with < 30%	380	
Parasitaemia (/μl)		
GM	34,044	30,400-33,664
Range	2009-1,194,285	
No with > 100,000 (/μl)	638 (23.2%)	
No. with >250,000 (/μl)	187 (6.8%)	
Gametocytaemia (/μl) GM	27 (6 - 4188)	
Duration of illness (d)	3.0 ± 1.4 (1-14)	2.9-3.0
Duration of vomiting (d)	1.3 ± 1.4 (1-9)	1.2-1.3

GM, geometric mean

### Treatment failures

Overall 57 of the 2,752 children (2.1%) had treatment failure by day 7, and this rose to 113/2,695 (4.2%) by day 14, and 62/796 (7.8%) by day 21 (χ^2 ^for trend = 155.8, P < 0.0001).

### Drug treatment and delay in parasite clearance

**Figure 1:**
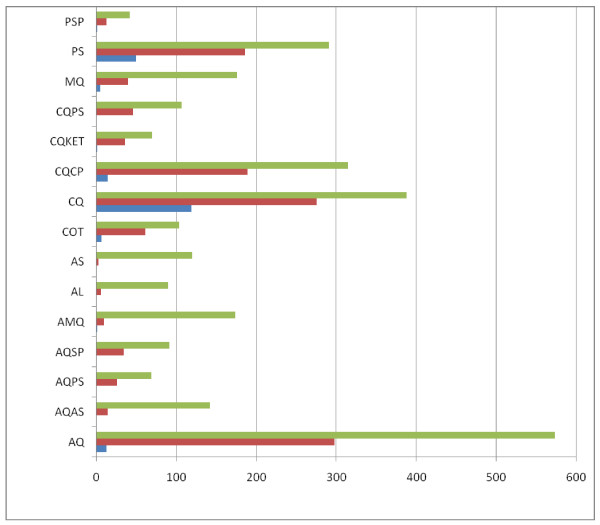
**Numbers of children with delay in parasite clearance (> 2 d) following treatment with antimalarial drugs**. Total enrolled (Green line), No. with parasite clearance > 2 d (Red line), No. with parasite clearance > 2 d who proceded to failure (Blue line). [see Table 1 for antimalarial drug abbreviations].

Overall, delay in parasite clearance occurred in 1237 of the 2752 children (45%) (Figure 1). The highest proportions of children showing delay in parasite clearance were found in those treated with CQ (70.8%), PS (63.9%), CQCP (60%), COT (58.6%) AQ (52%) CQKET (51.4%) CQPS (42%), AQPS (37.6%), AQSP (37.3%), PSP (30.9%), and MQ (22.7%). The proportions of children with delay in parasite clearance were significantly lower in those treated with AS (2.5%), AQAS (9.9%), AL (6.6%) and, AMQ (5.7%) when compared with the latter group above (χ^2 ^= 447.91, P < 0.0001). There was no significant difference in the proportions of children with delay in clearance in those treated with AS (3 of 120), AQAS (14 of 142), AL (6 of 90), and AMQ (10 of 174) (χ^2 ^= 6.1, df = 3, P = 0.10). The addition of AS to MQ (AMQ) significantly reduced the proportion of patients with delay in clearance (40 of 176 for MQ *v *10 of 174 for AMQ, χ^2 ^= 19.24, P = 0.0001). Similarly, the addition of AS to AQ (AQAS) significantly reduced the proportion of patients with delay in clearance (298 of 573 for AQ *v *14 of 142 for AQAS, χ^2 ^= 80.49, P < 0.0001).

### Temporal changes in parasite clearance time and proportion of children with delay in clearance in those treated with chloroquine, sulphadoxine-pyrimethamine and amodiaquine

**Figure 2:**
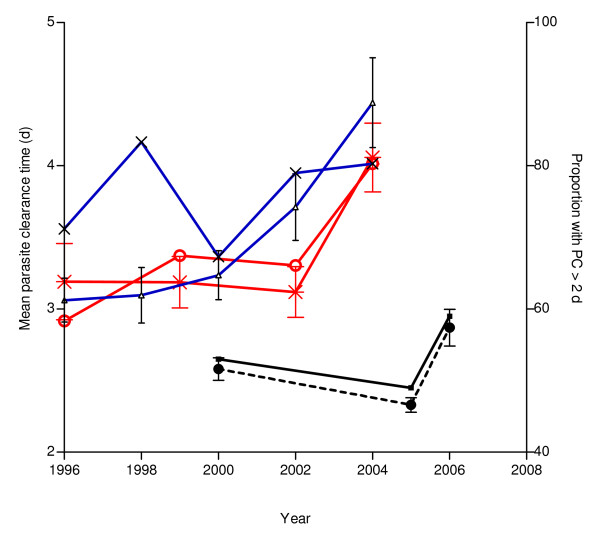
**Temporal changes in parasite clearance time (PCT) and proportion with delay in parasite clearance (PC > 2 d) in children treated with amodiaquine (AQ), chloroquine (CQ) or pyrimethamine sulphadoxine (PS) between 1996 and 2006**. [Proportion of children treated with AQ who had PC > 2 d (Black line), Proportion of children treated with CQ who had PC > 2 d (X), Proportion of children treated with PS who had PC > 2 d (O), PCT for AQ treated children (--•--), PCT for CQ treated children (Blue line), PCT for PS treated children (**+**)]. Bars represent standard deviation.

The changes in parasite clearance times (PCT) and the proportions of children with delay in clearance between 1996 and 2004, or between 2000 and 2006, in patients treated with CQ (n = 316), SP (n = 218), or AQ (n = 573) were evaluated. There was a significant increase in PCT from 3.08 ± 1.06 d (n = 53) in 1996 to 4.23 ± 2.08 d (n = 76) in 2004 (P < 0.0001), but there was no difference in proportion with delay in parasite clearance during the same period (71.7% in 1996 and 80.2% in 2004, χ^2 ^for trend = 1.36, P = 0.24) in those treated with CQ (Figure 2).

There was a significant increase in PCT from 3.19 ± 1.72 d (n = 48) in 1996 to 4.21 ± 1.84 d (n = 71) in 2004 (P = 0.0001) and a significant increase in proportion with delay in parasite clearance during the same period (58.3% in 1996 and 80.3% in 2004, χ^2 ^for trend = 5.99, P = 0.014) in those treated with SP (Figure 2).

There was also a significant increase in PCT from 2.58 ± 0.80 d (n = 104) in 2000 to 2.87 ± 1.39 d (n = 118) in 2006 (P = 0.0001), but there was no difference in proportion with delay in parasite clearance during the same period (53% in 2000 and 59% in 2006, χ^2 ^for trend = 0.81, P = 0.36) in those treated with AQ (Figure 2).

### Risk factors for delay in parasite clearance at enrolment

The following were found to be independent risk factors for delay in parasite clearance at enrolment (Table 3): age ≤ 2 years (Adjusted odds ratio [AOR] = 2.13, 95% confidence interval [CI] = 1.44-3.15, P < 0.0001), presence of fever (AOR = 1.33, 95% CI = 1.04-1.69, P = 0.019), parasitaemia >50,000/μl (AOR = 2.21, 95% CI = 1.77-2.75, P < 0.0001), and enrolment before year 2000 (AOR = 1.55, 95% CI = 1.22-1.96, P < 0.0001). A history of vomiting was associated with an increased risk of delay in clearance (crude odds ratio = 1.34, 95% CI = 1.07 -1.58, P = 0.009).

**Table 3:** Predictors of delay in parasite clearance at presentation in children with acute falciparum malaria

Variables	Number enrolled	PC >2 d	Crude OR (95% CI)	P Value	Adjusted OR (95% CI)	P Value
Age (years)						
>2	2446	1076	1		1	
≤ 2	267	140	1.40 (1.09 -1.80)	0.008	2.13 (1.44 -3.15)	<0.0001
						
Gender						
Female	1342	607	1			
Male	1240	557	0.98 (0.84 -1.15)	0.870	-	-
						
Fever*						
Absent	686	252	1		1	
Present	1743	842	1.6 (1.34 -1.93)	<0.0001	1.33 (1.04-1.69)	0.019
						
Duration of illness (d)						
≤ 3	2085	926	1			
> 3	521	246	1.12 (0.90 - 1.30)	0.225	-	-
						
Haematocrit (%)						
≥ 30	614	182	1			
<30	380	114	1.01 (0.76 - 1.34)	0.904	-	-
						
Parasitaemia (/μl blood)						
≤ 50,000	1607	634	1		1	
> 50,000	1145	603	1.70 (1.46 -1.99)	<0.0001	2.21 (1.77 - 2.75)	< 0.0001
						
Gametocytaemia						
Absent	2086	896	1			
Present	232	90	0.84 (0.63 - 1.10)	0.224	-	-
						
Vomiting						
No	1005	519	1		1	
Yes	665	387	1.34 (1.07-1.58)	0.009	1.21 (0.90-1.51)	0.089
						
Hepatomegaly						
Absent	471	237	1			
Present	798	430	1.54 (0.91 - 1.44)	0.219	-	-
						
Year of enrolment						
2000 onward	2165	872	1		1	
Before or 1999	587	365	2.43 (2.02 - 2.94)	0.0.00	1.55 (1.22-1.96)	< 0.0001

CI, confidence interval; OR, odd ratio; PC, parasite clearance, *Body temperature ≥ 37.5°C

### Risk factors for delay in parasite clearance following initiation of treatment

Following treatment, a body temperature ≥ 38°C and parasitaemia > 20,000/μl blood a day after treatment began, were independent risk factors for delay in clearance (Table 4). Non-artemisinin monotherapy was associated with delay in clearance.

**Table 4:** Predictors of delay in parasite clearance on day 1 after treatment in children with acute falciparum malaria

Variables	Number enrolled	PC >2 d	Crude OR (95% CI)	P Value	Adjusted OR (95% CI)	P Value
Axillary temperature (°C)						
<38.0	2326	1012	1		1	
≥ 38.0	228	139	2.02 (1.53 -2.67)	<0.0001	1.80 (1.30-2.50)	< 0.001
						
Parasitaemia (/μl blood)						
≤ 20,000	1328	463	1		1	
>20,000	683	467	5.25 (4.20 - 6.48)	<0.0001	5.13 (4.14 - 6.35)	< 0.001
						
Drug treatment *						
CQ	388	275	1	1	1	
AQ	573	298	0.44 (0.33 - 0.58)	<0.0001	0.79 (0.63 - 0.98)	< 0.03
AQAS	142	14	0.05 (0.03 - 0.08)	<0.0001	0.24 (0.10 - 0.57)	< 0.0001
AQPS	69	26	0.24 (0.14 - 0.42)	<0.0001	0.44 (0.26 - 0.73)	0.002
AQSP	91	34	0.25 (0.15 - 0.39)	<0.0001	0.43 (0.29 - 0.68)	< 0.0001
AMQ	174	10	0.03 (0.01 - 0.05)	<0.0001	0.04 (0.02 - 0.09)	< 0.0001
AL	90	6	0.03 (0.01 - 0.07)	<0.0001	0.05 (0.02 - 0.12)	< 0.0001
AS	120	3	0.01 (0.00 - 0.03)	<0.0001	0.02 (0.00 - 0.06)	< 0.0001
COT	104	61	0.58 (0.37 - 0.91)	0.017	1.03 (0.68 - 1.36)	0.877
CQCP	315	189	0.61 (0.45 - 0.84)	0.002	1.09 (0.83 - 1.42)	0.512
CQKET	70	36	0.43 (0.25 - 0.73)	0.001	0.77 (0.47 - 1.25)	0.029
CQPS	107	46	0.31 (0.19 - 0.48)	<0.0001	0.54 (0.36 - 0.82)	0.004
MQ	176	40	0.12 (0.08 - 0.18)	<0.0001	0.21 (0.15 - 0.31)	< 0.0001
SP	291	186	0.72 (0.52 - 1.00)	0.055	-	-
SPP	42	13	0.18 (0.09 - 0.37)	<0.0001	0.33 (0.17 -0.98)	0.001

CI, confidence interval; OR, odd ratio; PC, parasite clearance

* Values of OR represent chances of delay in parasite clearance

### Delay in parasite clearance and treatment failure

**Figure 3:**
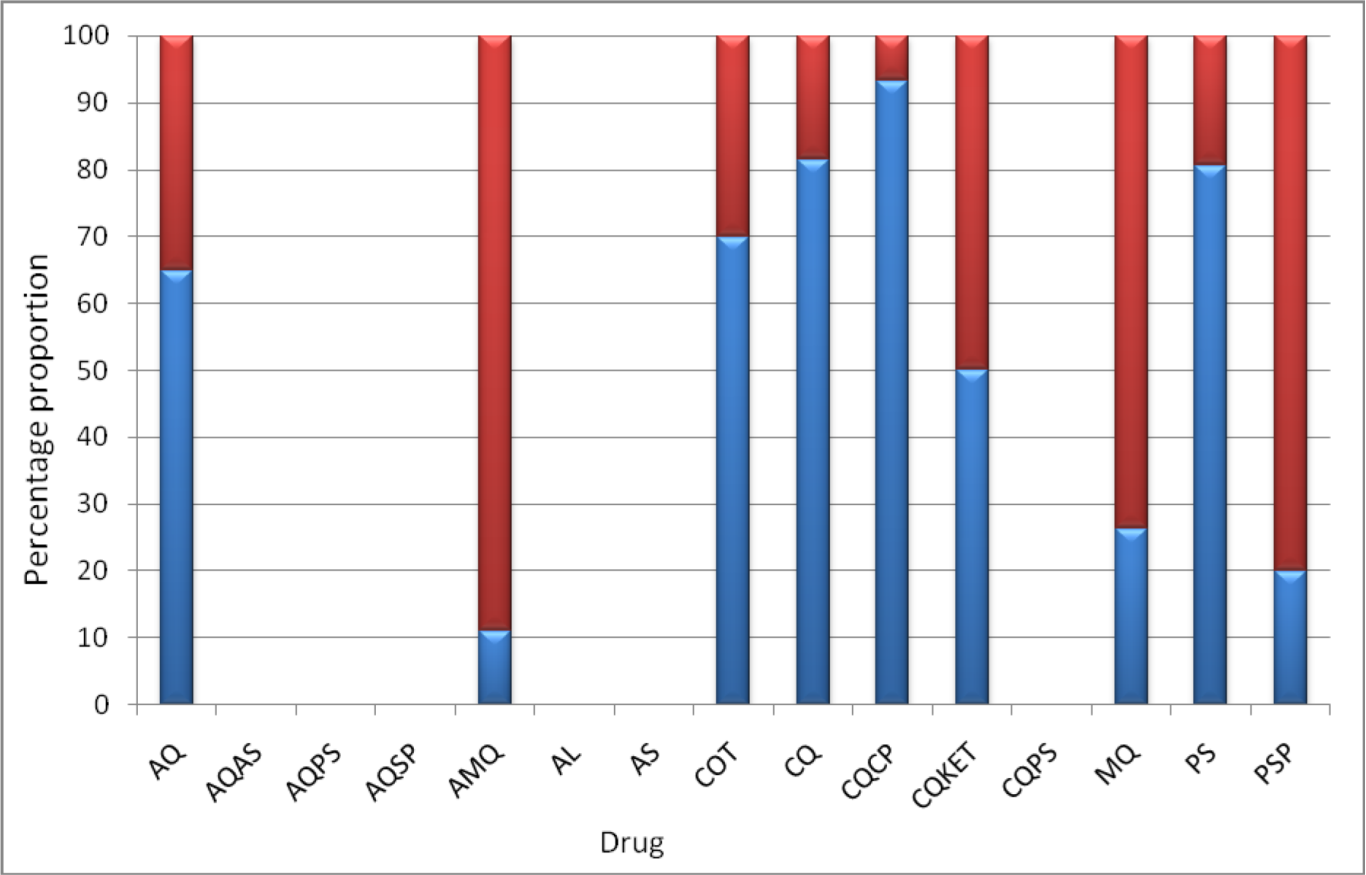
**Treatment failures following antimalarial drugs in children**. Total failure (Red triangle), children who previously had delay in parasite clearance (Blue triangle).

Of the 20, 146, 10, 15, 19, and 62 children who failed treatment with AQ, CQ, COT, CQCP, MQ, and PS, respectively, 13, 119, 7, 14, 5, and 50 children, respectively were those who previously had delay in parasite clearance (Figure 3). Overall, 291 children failed treatment. Of these, 211 (72%) had delay in parasite clearance. The latter represent 17% of the total children (1237) with delay in parasite clearance. Thus delay in clearance may be related to subsequent drug treatment failure.

### Gametocyte carriage

Overall 10% (282/2,752) of the children had patent gametocytaemia on presentation. In those treated with CQ gametocyte carriage at enrolment did not increase from 1996 (26%, 14/53) to 2003 (22% 14/62), χ^2 ^for trend = 0.417, P = 0.23. Similarly, in those treated with SP, gametocyte carriage at enrolment did not increase from 1996 (22%, 11/48) to 2003 (20% 15/72), χ^2 ^for trend = 0.075, P = 0.78. In those treated with AQ gametocyte carriage at enrolment also did not increase from 2000 (11.5%, 12/104) to 2006 (13% 16/120), χ^2 ^for trend = 0.55, P = 0.46.

**Figure 4:**
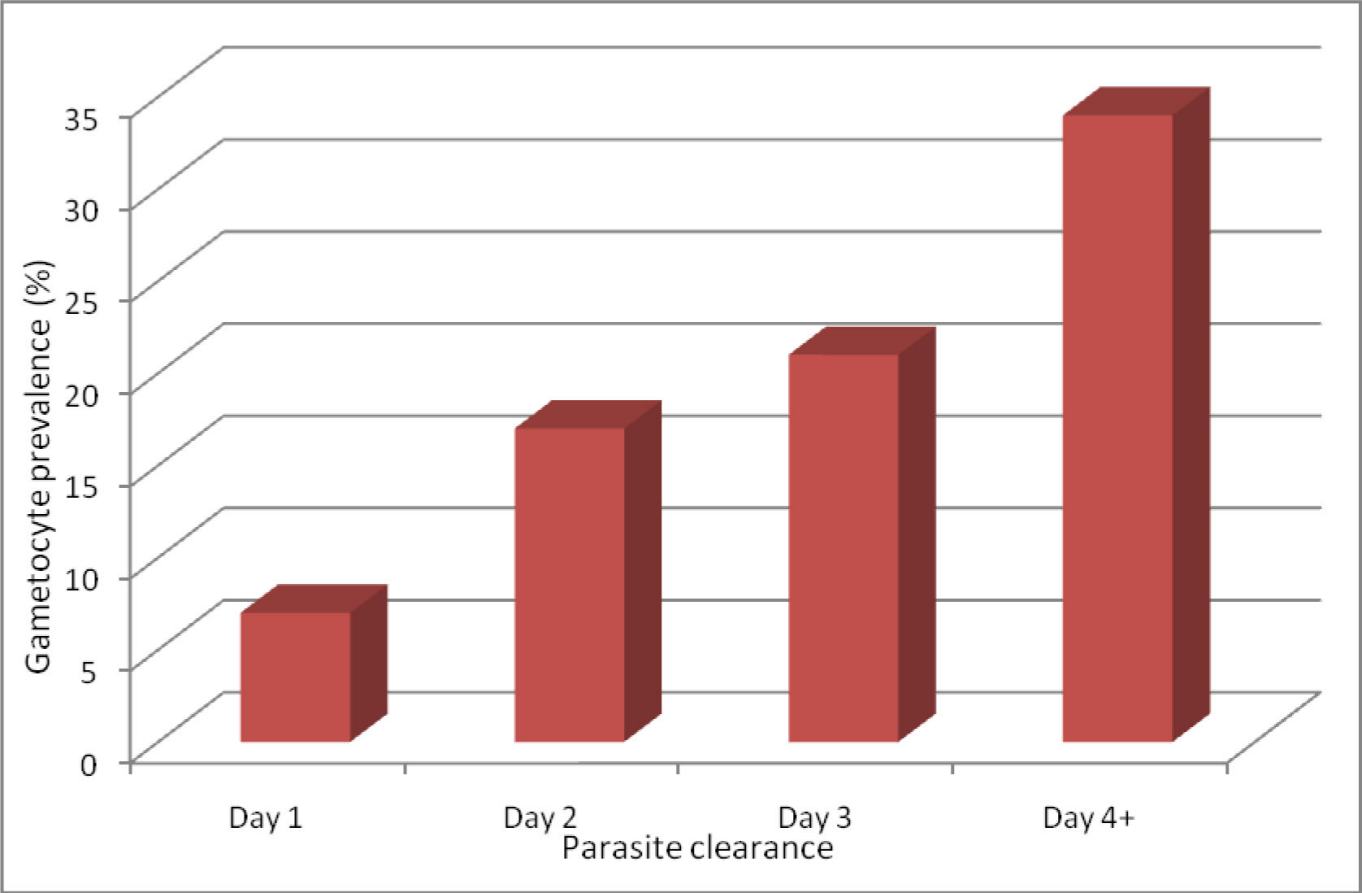
**Relationship between parasite clearance and gametocyte carriage**.

The majority of children without gametocytaemia at enrolment, who later developed gametocytaemia did so on day 7 (36%, 156/433). In those who did not carry gametocyte at enrolment, gametocyte carriage within two weeks of commencing therapy was related to parasite clearance time; it rose from 13% (188/1,374) among children who cleared their parasitaemia on day 1 or on day 2 to 24.5% (245/1,000) among those who cleared their parasitaemia on day 3 or on day 4 (χ^2 ^= 44.69, P < 0.0001) (Figure 4).

### *Pfmdr 1 *and *pfcrt *findings

A total of 260 isolates from 260 children were selected for genetic analysis of *pfmdr1/pfcrt *of which 256 were successfully determined. None of the 260 children was aged ≤ 2 years, but 137 children were aged 3 to < 5 years. Infections were classified as mutant for *pfmdr 1/pfcrt*, mixed, or wild type. The prevalence of the three categories were 51% (70/139), 36% (51/139) and 13% (15/139), respectively in 2000. In 2006, the corresponding prevalence was 50% (59/118), 36% (42/118) and 14% (17/118). In the 256 children, 13% (18/129), 4% (4/93), and 5% (2/34) with mutant, mixed, and wild alleles, respectively were treatment failures. The difference between these proportions was significant (χ^2 ^= 6.49, df = 2, P = 0.039). The proportions of 3 to < 5 year-olds with parasite clearance > 2 d in these groups were: 75% (48/64), 51% (28/54) and 63% (12/19), respectively for mutants, mixed and wild type. The difference in these proportions was significant (χ^2 ^= 6.84, df = 2, P = 0.032).

## Discussion

The emergence of drug resistance in *P. falciparum*, in particular to CQ and SP, in south-western Nigeria has been relatively slow but steady [12-14,25,26]. The aetiology of the treatment failure in children from this endemic area has been multifactorial including the selection of *Pfmdr*-1 gene following anti-malarial treatment [7,10]. The overall parasitological failure rate of over 40% with CQ and SP [25,26] clearly warrants discontinuing the use of these drugs as monotherapies or their inclusion as part of combination therapies. Thus, the recommended continuing use of SP in intermittent preventive therapy in pregnancy in this endemic area [27] should have little or no impact because of widespread clinical and molecular markers of resistance to this drug [28]. The latter is supported by a recent study (Gbotosho & Happi, personal communication), showing lack of protective value of SP in intermittent preventive therapy of malaria in a group of pregnant women in this endemic area.

Resistant infections at case management and community levels are difficult to manage and early identification and prompt treatment of patients at risk of subsequent treatment failure would improve patient care and community management of resistant infections. In the present study, a systematic evaluation of the pre-treatment and during-treatment factors contributing to delay in parasite clearance, the relationship between delay in parasite clearance and treatment failure, and effects of non-artemisinin and artemisinin mono- or combination therapy on parasite clearance in children were done. The results showed that delay in parasite clearance is multifactorial in origin with the host, parasite and drug factors contributing almost equally to delay in clearance and subsequent failure of treatment in those with delay in parasite clearance.

The age-and pretreatment parasite population size- dependent delay in parasite clearance are likely linked to treatment outcome by affecting both the immune-dependent ability to clear and the probability of survival of a subpopulation of asexual blood parasite stages, respectively. In cases of polyclonal infections, a common finding in this endemic area [7,29,30], this subpopulation may likely comprise drug-insensitive parasites [22,31,32]. Thus, these two phenomena are reminiscent of the age- and parasite density-dependent risks of treatment failure for many anti-malarial drugs [3,5,25,26,33,34]. Therefore, it would appear delay in parasite clearance is an intermediate step in the progression from drug-sensitive to drug insensitive outcome for many anti-malarials. This should be so because in uncomplicated malaria infections, there is less inhibition of parasite multiplication by unbound anti-malarial drug levels as resistance increases [31].

Interestingly, pre-treatment and intra-treatment elevated body temperatures were independent risk factors for delay in clearance. Fever is considered a crude measure of unspecific immune responsiveness [35]. If the findings are not due to chance, the presence of elevated body temperature a day after treatment began, coupled with a high parasite density should be sensitive clinical indicators of delay in response to anti-malarial drugs. Indeed, high parasite density on day 1 is a very sensitive indicator of delay in parasite clearance (Table 4). Again, these are reminiscent of the indicators of the risk factors for treatment failure for many anti-malarial drugs.

In general, progressive and significant elongations in parasite clearance times over time were striking features of treatment outcomes in children treated with CQ, and SP, the two most widely and longest used drugs in south-western Nigeria, and for AQ, a drug that is less frequently used, but is a partner drug for artesunate. These elongations were not accompanied by significant increases in proportions of patients with delay in parasite clearance during the same periods in those treated with CQ and AQ, suggesting similar mechanism(s) for development of resistance in the parasite to both drugs. In addition, the absence of increase in proportion with delay in clearance over time could have been due to the fact that these proportions were already above 50% when the studies began in 1996 and 2000, respectively for CQ and AQ. This would suggest that the 50% threshold may be the time to consider change in treatment policy at the community or national level in this endemic area.

Non-artemisinin monotherapy and non-artemisinin combination therapy were associated with significantly increased proportions of children with delay in clearance. These findings call for a new strategy to determine the maximum point in time at which to effect change in therapy in policy formulation with the widespread use of ACT in Africa. Perhaps this time point may be when 50% of a population of under-three to under-five year olds show delay in parasite clearance. This is best exemplified by the results from the children treated with amodiaquine, where over 50% showed a delay in clearance and 65% of those who were treatment failures were those with demonstrable delay in clearance. These findings contrast sharply with those found in children treated with artesunate and artemisinin-based combination therapy and strongly support the WHO recommendation of ACT [1] as first line treatment globally. However, it is likely that with increasing use of ACT on the African continent, significant increases in the proportion of children with delay in parasite clearance will be seen within a decade as has recently been demonstrated for artesunate-mefloquine use in north-western Thailand [36].

Delay in parasite clearance was associated with increased gametocyte carriage and, therefore, presumably with a potential for increased transmissibility of drug resistant phenotype. This is of public health import since delay in parasite clearance, by virtually all anti-malarial drugs, including ACT, is associated with an increased risk of gametocytaemia [36-38].

Over the study period the prevalence of *pfmdr1/pfcrt mutants *and mixed mutants/wild was above 50%, and with little or no increase between 2000 and 2006. Intriguingly, this was associated with increase in parasite clearance time in CQ and AQ-treated patients. Carriage of mutant parasites was associated with an increased risk of treatment failure. Additionally, the proportions of under five-year-olds with delay in parasite clearance increased with genotype classification suggesting that delay in parasite clearance was associated with drug resistance and treatment failure in young children. However, there are other factors that may affect susceptibility that were not evaluated in these children.

There is need to justify the definition of delay in clearance used in this study: one asexual cycle of the blood stage infection takes approximately 48 h. Thus after two days, the parasites appearing in blood and visible by microscopy when the patient presents, should be the one to appear in blood 48 h after treatment began if the drug does not kill all the parasites [31]. Thus the use of delay in clearance, in the opposite, and in a more practical way, is analogous to the parasite reduction ratio [22], but with an inclination for easy applicability and operational research.

## Conclusion

A better understanding of the multifactorial causes and mechanisms of delay in parasite clearance following anti-malarial treatment regimens can provide vital information for setting policy recommendations at case management, community, and national levels and prolonging the clinical life of the currently useful anti-malarials.

## Conflict of interests

The authors declare that they have no competing interests.

## Authors' contributions

AS led the design, conduct, data analysis and manuscript preparation. EOA was involved in manuscript preparation. GOG and CTH were involved in design, conduct, and preparation of the manuscript. AS was involved with data analysis. OAF, TMO and OSM were involved with conduct of study. All authors read and approved the manuscript.
